# Supplementary material for: Proteomic Analysis of the Protective Effect of Early Heat Exposure against Chronic Heat Stress in Broilers
Source: Animals (Basel). 2020 Dec 10;10(12):2365. doi: 10.3390/ani10122365 (PMC7764366; doi:10.3390/ani10122365)
Supplement: Supplementary file 1 [file animals-10-02365-s001.pdf]

Table S1. List of primer sequences for RT-qPCR.

Gene name	Uniprot ID	F/R	Sequence(5′-3′)	Product size
GOT1	P00504	F	CAAGCTGTCAAGCTGCTGTC	71
		R	CGTGGAGGAAAGCTAGCAAC	
OGDHL	E1BTL0	F	CCCTTCTCACTTGGAAGCAG	81
		R	CCTGCAGTATCCCCTCGATA	
UGT2A1	F1NMB3	F	GGAGCAAAGCACTTGAGACC	93
		R	GGCTGCACAGATGAACAAGA	
GART	P21872	F	GGAGATGGCTCGGACATTTA	90
		R	TTCTGCACATCCTTGAGCAC	
GSTT1L	E1BUB6	F	GTGCTACCGAGGAGCTGAAC	105
		R	CTACGAGGTCTGCCAAGGAG	
IARS	Q5ZKA2	F	GACAGGTTTCCTGGCATTGT	148
		R	GGGCTTGATGAACAACACCT	
RARS	Q5ZM11	F	TCATTGCTCACCTGCAAGAC	146
		R	CAGCACCACACATTGGTAGG	
GSS	F1NLE4	F	ACTGGATGTGGGTGAAGAGG	89
		R	CTCCTTCTCGCTGTGGTTTC	
CYP2D6	F1NJG4	F	AGGAGAAAGGAGGCAGAAGC	113
		R	TGTTGCTCCAAGATGACAGC	
GAPDH	P00356	F	GACGTGCAGCAGGAACACTA	112
		R	CTTGGACTTTGCCAGAGAGG	

Table S2. List of differentially expressed proteins during chronic heat stress.

name	Description	MW	score		PI
			CC	CH	
Down regulated by chronic heat stress					
A2M	Uncharacterized protein	158	1	0.35	6.62
A2ML4	Uncharacterized protein	163	1	0.09	6.37
ABCA8	Uncharacterized protein	185	1	0.43	7.08
ABCB1	Uncharacterized protein	152	1	0.47	8.43
ACOX2	Cluster of Acyl-coenzyme A oxidase	75	1	0.21	8
ACTN1	Alpha-actinin-1	102	1	0.37	5.55
ALDOC	Cluster of Fructose-bisphosphate aldolase	39	1	0.5	6.64
AMDHD1	Cluster of Uncharacterized protein	37	1	0.04	6.76
AMT	Aminomethyltransferase, mitochondrial	42	1	0.29	9.14
AP1B1	AP complex subunit beta	103	1	0.15	5.16
APOA1BP	NAD(P)H-hydrate epimerase	32	1	0.4	8.62
ARPC1A	Actin-related protein 2/3 complex subunit	42	1	0.34	8.31
ASS1	Argininosuccinate synthase	47	1	0.04	6.67
ATP2A2	Cluster of Calcium-transporting ATPase	114	1	0.22	5.36
ATP6V1A	Cluster of V-type proton ATPase catalytic subunit A	68	1	0.05	5.86
BCL2L14	Uncharacterized protein	40	1	0.41	5.19
C5H14orf166	Uncharacterized protein	28	1	0.44	6.44
CAPN11	Calpain-11	80	1	0.11	5.03
CCDC58	Uncharacterized protein	14	1	0.41	8.18
CFH	Uncharacterized protein	148	1	0.08	7.05
CHCHD3	MICOS complex subunit	27	1	0.35	8.97
CLTA	Clathrin light chain	24	1	0.21	4.5
CNBP	Cluster of Cellular nucleic acid-binding protein	15	1	0.26	6.6
CNN3	Calponin	37	1	0.25	6.62
COPE	Coatomer subunit epsilon	34	1	0.03	5.14
CRYM	Cluster of Uncharacterized protein	33	1	0.1	6.09
CS	Citrate synthase	60	1	0.34	8.91
CYFIP2	Cluster of Uncharacterized protein	138	1	0.43	-

CYP2AC2	Uncharacterized protein	57	1	0.08	9.17
CYP2D6	Cytochrome P450 CYP2D49	58	1	0.4	8.72
CYP4A22	Uncharacterized protein	59	1	0.45	8.25
DBNL	Uncharacterized protein	45	1	0.41	5.69
DCN	Decorin	40	1	0.26	8.32
DHFR	Dihydrofolate reductase	22	1	0.44	8.07
EIF3A	Cluster of Eukaryotic translation initiation factor 3 subunit A	143	1	0.37	6.96
EIF3B	Eukaryotic translation initiation factor 3 subunit B	85	1	0.26	5.15
EIF3G	Uncharacterized protein	29	1	0.33	7.66
EIF3I	Eukaryotic translation initiation factor 3 subunit I	37	1	0.34	5.64
EIF3K	Eukaryotic translation initiation factor 3 subunit K	27	1	0.04	5.01
EIF4E	Eukaryotic translation initiation factor 4E	25	1	0.22	5.95
EIF4G1	Uncharacterized protein	178	1	0.21	5.29
ENDOG	Uncharacterized protein	16	1	0.36	9.6
ENO3	Beta-enolase	47	1	0.07	7.61
FAAH	Cluster of Uncharacterized protein	64	1	0.41	6.21
FAHD2AL	Uncharacterized protein	38	1	0.35	9.77
FERMT2	Uncharacterized protein	81	1	0.15	6.51
FGB	Fibrinogen beta chain	55	1	0.31	7.36
FKBP8	Peptidylprolyl isomerase	44	1	0.03	4.89
GANC	Uncharacterized protein	105	1	0.42	6.37
GART	Trifunctional purine biosynthetic protein adenosine-3	107	1	0.17	7.58
GBP4L	Uncharacterized protein	70	1	0.04	5.91
GLO1	Lactoylglutathione lyase	21	1	0.25	6.55
GLOD4	Uncharacterized protein	33	1	0.42	6.25
GNB4	Uncharacterized protein	38	1	0.11	6.28
GOT1	Aspartate aminotransferase	46	1	0.13	8.12
GSS	Glutathione synthetase	52	1	0.07	5.88

HDLBP	Vigilin	142	1	0.43	7.25
HNRNPH2	Uncharacterized protein	43	1	0.13	6.76
HPX	Hemopexin	43	1	0.31	5.55
HSP90AB1	Heat shock cognate protein HSP 90-beta	80	1	0.12	5.34
HSPA4L	Uncharacterized protein	95	1	0.39	5.52
IDH3A	Isocitrate dehydrogenase [NAD] subunit, mitochondrial	40	1	0.19	6.54
KIF5B	Kinesin-like protein	110	1	0.32	6.44
KMO	Kynurenine 3-monooxygenase	55	1	0.44	8.44
KPNB1	Uncharacterized protein	120	1	0.37	6.06
KRT75L4	Cluster of Uncharacterized protein	70	1	0.38	7.2
KTN1	Kinectin	163	1	0.33	5.86
LETM1	Mitochondrial proton/calcium exchanger protein	86	1	0.06	6.71
LIMA1	Uncharacterized protein	85	1	0.2	5.95
LOC100857197	Uncharacterized protein	61	1	0.42	5.91
LOC107050352	Cluster of Eukaryotic translation initiation factor 5A	15	1	0.48	5.34
LOC107080643	Uncharacterized protein	27	1	0.09	8.02
LOC769704	Carboxylic ester hydrolase	37	1	0.41	5.35
LYZ	Lysozyme C	16	1	0.21	9.07
MARCKS	Myristoylated alanine-rich C-kinase substrate	28	1	0.49	4.4
MDN1	Uncharacterized protein	317	1	0.23	-
MYH9	Myosin-9	227	1	0.21	5.57
MYO1B	Myosin IB	125	1	0.26	9.2
NAPA	Cluster of Uncharacterized protein	29	1	0.08	5.25
NDUFB5	NADH:ubiquinone oxidoreductase subunit B5	21	1	0.36	9.13
NDUFS5	Uncharacterized protein	13	1	0.46	8.18
NDUFS7	Uncharacterized protein	24	1	0.42	9.89
NMT1	Glycylpeptide N-tetradecanoyltransferase	57	1	0.04	8
NOP56	Uncharacterized protein	60	1	0.09	7.62
NPEPPS	Uncharacterized protein	49	1	0.15	4.97
OIH	Ovoinhibitor	52	1	0.16	6.58
OTUB1	Ubiquitin thioesterase	31	1	0.04	5.1

PACSIN2	Protein kinase C and casein kinase substrate in neurons protein 2	56	1	0.44	5.3
PAFAH1B1	Lissencephaly-1 homolog	46	1	0.48	7.37
PARP1	Poly [ADP-ribose] polymerase	112	1	0.17	-
PDCD6IP	Programmed cell death 6 interacting protein	97	1	0.26	6.4
PDIA4	Uncharacterized protein	32	1	0.35	6.89
PGRMC2	Uncharacterized protein	22	1	0.02	5.38
PON2	Serum paraoxonase/arylesterase 2	39	1	0.06	5.34
PPP1CA	Serine/threonine-protein phosphatase	37	1	0.11	7.03
PPP1CC	Cluster of Serine/threonine-protein phosphatase	37	1	0.33	-
PSMA1	Proteasome subunit alpha type	29	1	0.16	6.54
PSMA3	Proteasome endopeptidase complex	26	1	0.04	5.15
PSMB1	Proteasome subunit beta	26	1	0.29	6.89
PSMD11	Uncharacterized protein	47	1	0.26	6.37
PTER	Phosphotriesterase related protein	39	1	0.17	6.58
PUF60	Uncharacterized protein	57	1	0.08	5
RAB5C	Ras-related protein Rab-5C	24	1	0.17	8.41
RAB8A	Ras-related protein Rab-8A	24	1	0.02	9.09
RDX	Radixin	69	1	0.49	6.37
RPIA	Uncharacterized protein	26	1	0.35	6.14
RPN2	Dolichyl-diphosphooligosaccharide--protein glycosyltransferase subunit 2	69	1	0.21	6.32
RPS19	Ribosomal protein S19	15	1	0.39	10.32
RPS21	40S ribosomal protein S21	9	1	0.5	8.5
RPS26	40S ribosomal protein S26	13	1	0.34	11
SAR1A	Uncharacterized protein	22	1	0.03	6.68
SCARB2	Uncharacterized protein	54	1	0.16	5.57
SLC9A3R2	Na(+)/H(+) exchange regulatory cofactor NHE-RF	39	1	0.4	7.87
SNX1	Uncharacterized protein	58	1	0.04	5.41
SORD	Cluster of Sorbitol dehydrogenase	38	1	0.34	7.39
SPIK5	Cluster of Uncharacterized protein	52	1	0.31	6.58
ST13	Hsc70-interacting protein	40	1	0.12	5.14

STT3A	Cluster of Uncharacterized protein	81	1	0.12	7.99
SULT1B1	Sulfotransferase family cytosolic 1B member 1	34	1	0.1	7.18
SUMO3	Small ubiquitin-related modifier	11	1	0.26	5.5
TARDBP	TAR DNA-binding protein 43	45	1	0.41	6.43
TF	Ovotransferrin	78	1	0.22	7.12
THNSL2	Uncharacterized protein	30	1	0.04	6.98
TTC38L	Uncharacterized protein	42	1	0.12	6.11
U2AF1	U2snRNP auxiliary factor small subunit	28	1	0.18	8.81
UBE2N	Uncharacterized protein	17	1	0.37	6.57
UGGT1	UDP-glucose glycoprotein glucosyltransferase 1	180	1	0.06	5.52
UROD	Uncharacterized protein	36	1	0.34	7.17
WDR1	WD repeat-containing protein 1	65	1	0.2	6.67
XDH	Xanthine dehydrogenase/oxidase	150	1	0.34	7.2
XYLB	Xylulokinase	58	1	0.03	6.54
YBX3	Uncharacterized protein	31	1	0.09	10.37
Up regulated by chronic heat stress					
APEH	Uncharacterized protein	82	1	2	6.13
PTBP1	Uncharacterized protein	60	1	2.01	9.32
NARS	Uncharacterized protein	64	1	2.02	6.04
NPC2	Uncharacterized protein	16	1	2.03	6.51
HIBADH	3-hydroxyisobutyrate dehydrogenase	35	1	2.03	8.32
UBAP2L	Uncharacterized protein	113	1	2.04	6.89
FLNB	Uncharacterized protein	284	1	2.04	5.69
GSTO1	Uncharacterized protein	27	1	2.04	7.44
PHYH	Uncharacterized protein	39	1	2.04	8.24
GALK1	Uncharacterized protein	42	1	2.06	6.09
HSD17B7	Uncharacterized protein	34	1	2.06	7.37
RTCB	tRNA-splicing ligase RtcB homolog	55	1	2.06	7.24
SPTAN1	Cluster of Spectrin alpha chain, non-erythrocytic 1	286	1	2.06	5.36
HDHD2	Uncharacterized protein	28	1	2.06	6.87
HKDC1	Uncharacterized protein	102	1	2.08	7.81
ARCN1	Coatomer subunit delta	57	1	2.08	6.07

CRYL1	Uncharacterized protein	35	1	2.08	6.79
RARS	Arginine--tRNA ligase, cytoplasmic	75	1	2.08	6.8
SEC61A1	Uncharacterized protein	52	1	2.08	8.06
DNTTIP2	Uncharacterized protein	30	1	2.08	5.27
RPS27	40S ribosomal protein S27	9 k	1	2.09	9.45
cpsmb7	Cluster of Proteasome subunit beta	30	1	2.09	6.4
EHD3	Uncharacterized protein	61	1	2.09	6.51
SNX3	Uncharacterized protein	13	1	2.09	8.87
TIMM8A	Uncharacterized protein	11	1	2.1	5.17
RAB7A	RAB7A, member RAS oncogene family	24	1	2.1	6.7
CYP4V2	Uncharacterized protein	62	1	2.11	6.64
GLUD1	Glutamate dehydrogenase 1, mitochondrial	56	1	2.11	8.28
RAB8B	Uncharacterized protein	24	1	2.11	9.06
OGDH	Uncharacterized protein	115	1	2.12	6.96
AKR1A1	Cluster of Alcohol dehydrogenase [NADP(+)]	37	1	2.12	-
CYP1A2	Cluster of Cytochrome P450	60	1	2.13	8.07
YWHAQ	14-3-3 protein theta	28	1	2.13	4.78
SSR4	Uncharacterized protein	30	1	2.13	7.36
CHORDC1	Cysteine and histidine-rich domain-containing protein 1	37	1	2.13	7.24
SLCO1A2	Solute carrier organic anion transporter family member	73	1	2.13	7.3
CLUH	Clustered mitochondria protein homolog	151	1	2.14	6.06
SLC27A4	Uncharacterized protein	89	1	2.14	9.04
APMAP	Adipocyte plasma membrane-associated protein	46	1	2.14	6.46
SUB1	Activated RNA polymerase II transcriptional coactivator p15	14	1	2.14	-
NMT2	Glycylpeptide N-tetradecanoyltransferase	56	1	2.14	9.28
NUDC	Nuclear migration protein nudC	39	1	2.15	6.83
VPS29	Vacuolar protein sorting-associated protein 29	21	1	2.15	7.62
CCT8	T-complex protein 1 subunit theta	59	1	2.15	5.53
DECR2	Uncharacterized protein	32	1	2.18	9.07
CALM2	Cluster of Uncharacterized protein	16	1	2.18	4.25

PSMC6	Uncharacterized protein	46	1	2.18	7.2
SLC16A1	Solute carrier family 16 member 1	54	1	2.25	8.31
PPIF	Peptidyl-prolyl cis-trans isomerase	22	1	2.32	8.98
GRPEL1	GrpE protein homolog	25	1	2.41	7.49
ABAT	Uncharacterized protein	55	1	2.41	8.12
CORO1C	Cluster of Coronin	64	1	2.41	6.67
SNAP23	Synaptosomal-associated protein	24	1	2.5	5.01
SARS	Uncharacterized protein	71	1	2.52	8.92
LOC415664	Uncharacterized protein	24	1	2.6	9
RPS27L	40S ribosomal protein S27	10	1	2.65	9.52
DYL1	Dynein light chain	10	1	2.73	7.44
DMGDH	Uncharacterized protein	80	1	2.74	6.65
CD320	Uncharacterized protein	15	1	2.76	10.33
NDUFA2	NADH dehydrogenase [ubiquinone] 1 alpha subcomplex subunit 2	11	1	2.77	9.79
IARS	Uncharacterized protein	147	1	2.92	6.49
PDK3	Uncharacterized protein	44	1	2.92	8.97
GOT1	Aspartate aminotransferase, cytoplasmic	46	1	2.94	8.12
H2B-VII	Histone H2B 7	14	1	2.94	10.32
SGTA	Uncharacterized protein	34	1	2.96	4.82
SEC24A	Cluster of Uncharacterized protein	120	1	2.97	7.94
BID	BH3-interacting domain death agonist	22	1	2.97	4.96
PCCA	Uncharacterized protein	79	1	3	7.77
HNRNPA1	Uncharacterized protein	36	1	3.08	9.48
IGF2BP2	Uncharacterized protein	66	1	3.15	8.95
AP2M1	AP-2 complex subunit mu	50	1	3.16	9.54
LYPLA2	Uncharacterized protein	25	1	3.16	7.47
CYP4B7	Uncharacterized protein	58	1	3.17	8.65
TMEM30A	Cell cycle control protein 50A	41	1	3.18	8.31
CAPZB	F-actin-capping protein subunit beta isoforms 1 and 2	30	1	3.19	8.02
ASMTL	Uncharacterized protein	69	1	3.19	6.39
YARS	Tyrosine--tRNA ligase	60	1	3.19	6.65

SDSL	Uncharacterized protein	34	1	3.2	7.24
COTL1	ADF actin binding protein	16	1	3.21	5.44
TPM1	Tropomyosin alpha-1 chain	19	1	3.23	4.77
OGDHL	Uncharacterized protein	115	1	3.25	-
TOMM70	Uncharacterized protein	67	1	3.34	6.43
TXN	Thioredoxin	12	1	3.43	5.25
FTH	Ferritin heavy chain	21	1	3.77	6.21
ALDH18A1	Delta-1-pyrroline-5-carboxylate synthase	88	1	3.81	7.03
chPKCI	Cluster of Protein kinase C inhibitor	14	1	3.93	6.79
ES1ML1	Cluster of Uncharacterized protein	27	1	4.2	6.62
HP1BP3	Heterochromatin protein 1-binding protein 3	62	1	4.22	9.33
MTHFS	5-formyltetrahydrofolate cyclo-ligase	22	1	4.22	8.13
TIMM44	Mitochondrial import inner membrane translocase subunit TIM44	51	1	4.23	8.12
CHCHD6	Cluster of MICOS complex subunit	28	1	4.28	6.32
MRI1	Uncharacterized protein	34	1	4.29	6.8
GSTT1L	Uncharacterized protein	28	1	4.29	7.03
ATPIF1	Uncharacterized protein	13	1	4.32	9.6
RAP1A	Uncharacterized protein	21	1	4.33	6.67
LOC107080643	Uncharacterized protein	27	1	4.67	8.02
HSPA9	Stress-70 protein, mitochondrial	73	1	4.82	6.43
INF2	Uncharacterized protein	144	1	4.96	5.68
GSPT1	Uncharacterized protein	68	1	5.13	5.14
ACSS1L	Acetyl-coA synthetase-2like, mitochondrial isoform X1	58	1	5.39	6.6
ACSM5	Uncharacterized protein	65	1	5.47	8.1
ACAD6L	Uncharacterized protein	33	1	5.85	6.9
SEC24A	Uncharacterized protein	120	1	5.85	7.94
ATP1A1	Sodium/potassium-transporting ATPase subunit alpha	113	1	6.28	5.53
TARS	Uncharacterized protein	91	1	6.43	8.07
CYP2C23b	Uncharacterized protein	56	1	6.45	6.67
CRIP2	Uncharacterized protein	26	1	6.46	9.2

VNN1	Cluster of Uncharacterized protein	57	1	6.66	5.41
QARS	Cluster of Uncharacterized protein	96	1	7.57	7.17
ACAD11	Acyl-CoA dehydrogenase family member 11	87	1	8.29	8.15
HIBCH	3-hydroxyisobutyryl-CoA hydrolase, mitochondrial	43	1	8.49	8.44
DHTKD1	Uncharacterized protein	103	1	8.65	6.79
GART	Trifunctional purine biosynthetic protein adenosine-3	107	1	9.67	7.58
ACAD9	Uncharacterized protein	37	1	9.75	8.76
HMGCL	Hydroxymethylglutaryl-CoA lyase, mitochondrial	34	1	10.26	7.88
AKR1B10L4	Uncharacterized protein	36	1	10.32	7.11
DYNC1I2	Uncharacterized protein	71	1	10.39	5.22
WDR1	WD repeat-containing protein 1	67	1	10.56	6.67
RGN	Regucalcin	33	1	10.9	6.07
tcp-1	T-complex protein 1 subunit delta	58	1	11.99	6.57
YWHAH	Tyrosine 3-monooxygenase/tryptophan 5-monooxygenase activation protein eta	28	1	13.21	4.89
SRSF7	Cluster of Uncharacterized protein	28	1	13.35	11.8
CBR4	Cluster of Uncharacterized protein	25	1	13.75	8.94
GSTAL3	Uncharacterized protein	26	1	13.86	9.03
GPI	Uncharacterized protein	26	1	14.09	8.13
RBP4	Retinol-binding protein 4	23	1	16.2	6.34
PSMD4	Uncharacterized protein	41	1	17.39	4.79
RAB6A	Cluster of Ras-related protein Rab-6A	20	1	18.24	5.48
PCYT2	Cluster of Uncharacterized protein	40	1	18.41	6.58
ETFA	Uncharacterized protein	34	1	19.93	7.01
ACP1	Cluster of Low molecular weight phosphotyrosine protein phosphatase	18	1	20.07	7.2
GSR	Glutathione reductase	50	1	21.09	7.52
CALM1	Uncharacterized protein	16	1	23.16	4.41
UGT2A1	UDP-glucuronosyltransferase	61	1	24.35	7.01
ATIC	Bifunctional purine biosynthesis protein PURH	69	1	25.5	8.18

FKBP3	Peptidylprolyl isomerase	26	1	26.39	-
CHDSD	Uncharacterized protein	40	1	34.27	7.56
CTSA	Carboxypeptidase	53	1	34.27	6.58
SNRPD2	Cluster of Small nuclear ribonucleoprotein Sm D2	20	1	36.81	10.04
RPL35A	Uncharacterized protein	12	1	61.13	11.05
GIMAP5	Uncharacterized protein	29	1	63.03	7.93
TXN2	Uncharacterized protein	16	1	64.63	8.84
CRYBA2	Beta-crystallin A2	23	1	83.2	6.68
FAM162A	Uncharacterized protein	17	1	85.75	10.24
ISG12(2)	Putative ISG12(2) protein	10	1	89.96	10.17

Table S3. List of differentially expressed proteins of positive effected by early heat exposure

No	UniProt	Description	Name	MW (kDa)	Score		
					CC	CH	HH
Low expressed by chronic heat stress							
1	F1NJG4	Cytochrome P450 CYP2D49	CYP2D6	58	1	0.40	0.80
2	F1NBI2	Uroporphyrinogen_deCOase domain-containing protein	UROD	36	1	0.34	0.70
3	F1NK40	Uncharacterized protein	A2ML4	163	1	0.09	1.18
4	P05094	Alpha-actinin-1 (Alpha-actinin cytoskeletal isoform) (F-actin cross-linking protein) (Non-muscle alpha-actinin-1)	ACTN1	102	1	0.37	0.88
5	F1P298	Amidohydro-rel domain-containing protein	AMDHD1	37	1	0.04	0.85
6	P28337	Aminomethyltransferase, mitochondrial, EC 2.1.2.10 (Glycine cleavage system T protein, GCVT)	AMT	42	1	0.29	0.94
7	-	Calpain-11	CAPN11	80	1	0.11	0.69
8	Q5ZHR7	Clathrin light chain	CLTA	24	1	0.21	0.83
9	A0A1L1RWF6	Calponin	CNN3	37	1	0.25	0.71
10	A0A1D5PLS2	Citrate synthase	CS	60	1	0.34	0.93
11	A0A1L1RWI4	Uncharacterized protein	CYP2AC2	57	1	0.08	0.68
12	A0A3Q2U335	ADF-H domain-containing protein	DBNL	45	1	0.41	0.98
13	P28675	Decorin (Bone proteoglycan II) (PG-S2)	DCN	40	1	0.26	1.06
14	A0A3Q3AA40	Eukaryotic translation initiation factor 3 subunit G, eIF3g (Eukaryotic translation initiation factor 3 RNA-binding subunit,	EIF3G	29	1	0.33	0.90

		eIF-3 RNA-binding subunit) (Eukaryotic translation initiation factor 3 subunit 4)					
15	A0A1D5P5T1	Eukaryotic translation initiation factor 3 subunit K, eIF3k (Eukaryotic translation initiation factor 3 subunit 12) (eIF-3 p25)	EIF3K	27	1	0.04	0.73
16	A0A3Q2TU97	Eukaryotic translation initiation factor 4E	EIF4E	25	1	0.22	1.16
17	A0A1D5PU09	PH domain-containing protein	FERMT2	81	1	0.15	0.83
18	E1C6R4	Peptidylprolyl isomerase, EC 5.2.1.8	FKBP8	44	1	0.03	0.69
19	P21872	Trifunctional purine biosynthetic protein adenosine-3 [Includes: Phosphoribosylamine--glycine ligase, EC 6.3.4.13 (Glycinamide ribonucleotide synthetase, GARS) (Phosphoribosylglycinamide synthetase)	GART	107	1	0.17	0.93
20	A0A1D5PZ32	GB1/RHD3-type G domain-containing protein	GBP4L	70	1	0.04	1.13
21	F1NLE4	Glutathione synthetase, GSH-S, EC 6.3.2.3	GSS	52	1	0.07	0.68
22	P20057	Hemopexin	HPX	43	1	0.31	0.83
23	A0A1D5P1W7	Importin N-terminal domain-containing protein	KPNB1	120	1	0.37	0.88
24	Q5ZK33	Mitochondrial proton/calcium exchanger protein (Leucine zipper-EF-hand-containing transmembrane protein 1)	LETM1	86	1	0.06	0.71
25	A0A3Q3B025	LIM zinc-binding domain-containing protein	LIMA1	85	1	0.20	0.56
26	A0A1D5PMD9	Carboxylic ester hydrolase, EC 3.1.1.-	LOC769704	59	1	0.38	1.02
27	P00698	Lysozyme C, EC 3.2.1.17 (1,4-beta-N-acetylmuramidase C) (Allergen Gal d IV) (allergen Gal d 4)	LYZ	16	1	0.21	0.86

28	P14105	Myosin-9	MYH9	227	1	0.21	0.46
29	F1NTJ5	Myosin IB	MYO1B	125	1	0.26	0.73
30	A0A1L1RKT0	Glycylpeptide N-tetradecanoyltransferase, EC 2.3.1.97	NMT1	57	1	0.04	0.34
31	O13154	Protein kinase C and casein kinase substrate in neurons protein 2 (Focal adhesion protein of 52 kDa, FAP52)	PACSIN2	56	1	0.44	0.99
32	A0A1D5PLK6	BRO1 domain-containing protein	PDCD6IP	97	1	0.26	0.72
33	Q5ZLX0	Cytochrome b5 heme-binding domain-containing protein	PGRMC2	22	1	0.02	0.48
34	O42265	Proteasome subunit alpha type-1, EC 3.4.25.1 (Macropain subunit C2) (Multicatalytic endopeptidase complex subunit C2) (Proteasome component C2)	PSMA1	29	1	0.16	0.64
35	E1BWG7	Phosphotriesterase related protein	PTER	39	1	0.17	0.94
36	A0A1D5PNR0	RNA-binding protein 8A	RBM8A	19	1	0.02	0.94
37	A0A1D5PDV6	Ribosomal protein S19	RPS19	15	1	0.39	0.86
38	Q5ZM66	40S ribosomal protein S26	RPS26	13	1	0.34	0.93
39	A0A1L1S0C5	PX domain-containing protein	SNX1	58	1	0.04	0.73
40	P0DMQ6	Sorbitol dehydrogenase, SDH, EC 1.1.1.- (Polyol dehydrogenase)	SORD	38	1	0.34	1.17
41	A0A1L1RY95	UBIQUITIN_CONJUGAT_2 domain-containing protein	UBE2N	17	1	0.37	1.14
42	A0A1D5NZ55	UDP-glucose glycoprotein glucosyltransferase 1	UGGT1	180	1	0.06	0.18

High expressed by chronic heat stress

43	F1NGM0	Uncharacterized protein	EHD3	61	1	2.09	0.84
44	F1NNH9	TPR_REGION domain-containing protein	TOMM70	67	1	3.34	0.90
45	Q5ZHT1	Acyl-CoA dehydrogenase family member 11	ACAD11	87	1	8.29	1.16
46	F1NEF6	Uncharacterized protein	ACAD9	37	1	9.75	1.16
47	Q5ZKG5	Low molecular weight phosphotyrosine protein phosphatase	ACP1	18	1	20.07	1.16
48	E1BZT9	Acetyl-coA synthetase-2like, mitochondrial isoform X1	ACSS1L	58	1	5.39	1.43
49	-	Uncharacterized protein	AKR1B10L4	36	1	10.32	1.16
50	P09572	Sodium/potassium-transporting ATPase subunit alpha-1, Na(+)/K(+) ATPase alpha-1 subunit	ATP1A1	113	1	6.28	1.16
51	A0A1D5PBD2	Uncharacterized protein	ATPIF1	13	1	4.32	1.43
52	A0A3Q3AZM2	Uncharacterized protein	CALM1	16	1	23.16	1.16
53	A9CP13	D-serine dehydratase	CHDSD	40	1	34.27	1.16
54	Q9I882	Protein kinase C inhibitor	chPKCI	14	1	3.93	1.33
55	A0A1D5PCT4	Coronin	CORO1C	64	1	2.41	0.95
56	P55164	Beta-crystallin A2	CRYBA2	23	1	83.20	1.16
57	A0A1L1RKJ5	Carboxypeptidase, EC 3.4.16.-	CTSA	53	1	34.27	9.25
58	Q5F412	Dynein light chain	DYL1	10	1	2.73	1.15
59	F1N9U8	ETF domain-containing protein	ETFA	34	1	19.93	1.16
60	F1NHG6	Uncharacterized protein	FAM162A	17	1	85.75	1.16
61	A0A1D5P3I6	Peptidylprolyl isomerase, EC 5.2.1.8	FKBP3	26	1	26.39	1.16

62	A0A1D5NYG3	Uncharacterized protein	FLNB	284	1	2.03	0.96
63	P21872	Trifunctional purine biosynthetic protein adenosine-3 [Includes: Phosphoribosylamine--glycine ligase	GART	107	1	9.67	1.16
64	P00504	Aspartate aminotransferase, cytoplasmic, cAspAT	GOT1	46	1	2.94	1.33
65	F1NIJ6	Glucose-6-phosphate isomerase	GPI	26	1	14.09	1.16
66	-	Uncharacterized protein	GSPT1	68	1	5.13	1.16
67	A0A1D5P338	Glutathione reductase	GSR	50	1	21.09	9.25
68	F1NQS2	Uncharacterized protein	GSTAL3	26	1	13.86	1.16
69	E1BUB6	Uncharacterized protein	GSTT1L	28	1	4.29	1.45
70	E1BRU7	Uncharacterized protein	HKDC1	102	1	2.08	0.94
71	P35915	Hydroxymethylglutaryl-CoA lyase, mitochondrial	HMGCL	34	1	10.26	1.16
72	Q5ZM98	Stress-70 protein, mitochondrial	HSPA9	73	1	4.82	1.16
73	Q5ZKA2	Isoleucine--tRNA ligase, mitochondrial	IARS	147	1	2.92	1.36
74	E1BWB9	Uncharacterized protein	INF2	144	1	4.96	1.16
75	Q6IEC5	Putative ISG12(2) protein	ISG12(2)	10	1	89.96	40.86
76	A0A1D5PAF0	Uncharacterized protein	LOC107080643	27	1	4.67	1.05
77	E1BRI5	Abhydrolase_2 domain-containing protein	LYPLA2	25	1	3.16	1.46
78	E1BTL0	Transket_pyr domain-containing protein	OGDHL	115	1	3.25	1.16
79	F1P0M2	Uncharacterized protein	PCCA	79	1	3.00	0.95
80	A0A1L1RW22	CTP_transf_like domain-containing protein	PCYT2	40	1	18.41	1.16
81	Q5ZLT2	Protein-serine/threonine kinase	PDK3	44	1	2.92	1.30

82	A0A1L1RXJ1	Uncharacterized protein	QARS	96	1	7.57	1.16
83	E1C0F3	Uncharacterized protein	RAB7A	24	1	2.10	0.96
84	Q5ZM11	Arginine--tRNA ligase, cytoplasmic	RARS	75	1	2.08	0.94
85	P41263	Retinol-binding protein 4	RBP4	23	1	16.20	1.16
86	E1BSA7	Uncharacterized protein	SEC24A	120	1	5.85	1.16
87	A0A1D5NVD4	Uncharacterized protein	SRSF7	28	1	13.35	1.16
88	A0A1D6UPQ3	AA_TRNA_LIGASE_II domain-containing protein	TARS	91	1	6.43	1.43
89	A0A1L1RMM0	Uncharacterized protein	tcp-1	58	1	11.99	1.16
90	F1NU71	Mitochondrial import inner membrane translocase subunit TIM44	TIMM44	51	1	4.23	1.42
91	P08629	Thioredoxin, Trx	TXN	12	1	3.43	1.70
92	A0A1D5PWT4	Thioredoxin domain-containing protein	TXN2	16	1	64.63	1.16
93	-	Uncharacterized protein	UGT2A1	61	1	24.35	8.10
94	A0A1D5PEU7	CN hydrolase domain-containing protein	VNN1	57	1	6.66	1.16
95	O93277	WD repeat-containing protein 1	WDR1	67	1	10.56	1.16

Table S4. List of Gene Ontology terms.

GO ID	Description	p-value	No,	Genes
Biological process				
0006662	glycerol ether metabolic process	0.0015	2	TXN, TXN2
0018904	ether metabolic process	0.0018	2	TXN, TXN2
0006749	glutathione metabolic process	0.00037	3	GSS, GSR, GSTAL3
0045454	cell redox homeostasis	0.00062	3	GSR, TXN, TXN2
0071466	cellular response to xenobiotic stimulus	0.0019	3	CYP2D6, CYP2AC2, EIF4E
0006790	sulfur compound metabolic process	0.0011	5	GSS, GSR, GSTAL3, HSPA9, PDK3
0051186	cofactor metabolic process	0.00029	7	GSS, GOT1, GSR, GSTAL3, HKDC1, HSPA9, PDK3
0030036	actin cytoskeleton organization	0.0019	7	ACTN1, LIMA1, MYH9, MYO1B, PACSIN2, FLNB, WDR1
0030029	actin filament-based process	0.0031	7	ACTN1, LIMA1, MYO1B, PACSIN2, FLNB, WDR1
0044281	small molecule metabolic process	0.00057	13	CYP2D6, CYP2AC2, GART, NMT1, UGGT1, GOT1, HKDC1, HMGCL, IARS, PDK3, RARS, TXN, TXN2
Cellular component				
0s042641	actomyosin	1.36E-08	6	ACTN1, FERMT2, LIMA1, MYH9, FLNB, WDR1
0001725	stress fiber	4.54E-07	5	ACTN1, FERMT2, LIMA1, MYH9, FLNB
0097517	contractile actin filament bundle	4.54E-07	5	ACTN1, FERMT2, LIMA1, MYH9, FLNB
0032432	actin filament bundle	1.17E-06	5	ACTN1, FERMT2, LIMA1, MYH9, FLNB

0005925	focal adhesion	1.31E-05	5	ACTN1, FERMT2, LIMA1, PACSIN2, FLNB
0005924	cell-substrate adherens junction	1.55E-05	5	ACTN1, FERMT2, LIMA1, PACSIN2, FLNB
0030055	cell-substrate junction	1.97E-05	5	ACTN1, FERMT2, LIMA1, PACSIN2, FLNB
0001726	ruffle	0.00015	4	ACTN1, LIMA1, MYH9, PACSIN2
0005912	adherens junction	0.00025	5	ACTN1, FERMT2, LIMA1, PACSIN2, FLNB
0015629	actin cytoskeleton	4.31E-05	7	ACTN1, FERMT2, LIMA1, MYH9, MYO1B, FLNB, WDR1
Molecular function				
0015037	peptide disulfide oxidoreductase activity	0.0011	2	GSR, TXN
0000146	microfilament motor activity	0.0011	2	MYH9, MYO1B
0015035	protein disulfide oxidoreductase activity	0.0029	2	TXN, TXN2
0015036	disulfide oxidoreductase activity	0.00041	3	GSR, TXN, TXN2
0016667	oxidoreductase activity, acting on a sulfur group of donors	0.001	3	GSR, TXN, TXN2
0051015	actin filament binding	3.32E-05	6	ACTN1, FERMT2, LIMA1, MYH9, MYO1B, WDR1
0016874	ligase activity	0.00019	5	GART, GSS, IARS, PCCA, RARS
0003779	actin binding	0.00013	7	ACTN1, FERMT2, LIMA1, MYH9, MYO1B, FLNB, WDR1
0008092	cytoskeletal protein binding	0.0048	8	ACTN1, FERMT2, LIMA1, MYH9, MYO1B, PACSIN2, FLNB, WDR1
0044877	protein-containing complex binding	0.0048	8	ACTN1, DCN, FERMT2, LETM1, LIMA1, MYH9, MYO1B, WDR1
